# Lithium for schizophrenia: supporting evidence from a 12-year, nationwide health insurance database and from Akt1-deficient mouse and cellular models

**DOI:** 10.1038/s41598-019-57340-8

**Published:** 2020-01-20

**Authors:** Da-Zhong Luo, Chia-Yuan Chang, Tsung-Ren Huang, Vincent Studer, Tsu-Wei Wang, Wen-Sung Lai

**Affiliations:** 1 Department of Psychology, National Taiwan University, Taipei, Taiwan; 2 Interdisciplinary Institute for Neuroscience, University of Bordeaux, F-33077 Bordeaux, France; 3 French National Center for Scientific Research (CNRS), UMR 5297, F-33077 Bordeaux, France; 4 Department of Life Science, National Taiwan Normal University, Taipei, Taiwan; 5 Graduate Institute of Brain and Mind Sciences, National Taiwan University, Taipei, Taiwan; 6 Neurobiology and Cognitive Science Center, National Taiwan University, Taipei, Taiwan

**Keywords:** Schizophrenia, Schizophrenia

## Abstract

Accumulating evidence suggests AKT1 and DRD2-AKT-GSK3 signaling involvement in schizophrenia. AKT1 activity is also required for lithium, a GSK3 inhibitor, to modulate mood-related behaviors. Notably, GSK3 inhibitor significantly alleviates behavioral deficits in *Akt1*^*−/−*^ female mice, whereas typical/atypical antipsychotics have no effect. In agreement with adjunctive therapy with lithium in treating schizophrenia, our data mining indicated that the average utilization rates of lithium in the Taiwan National Health Insurance Research Database from 2002 to 2013 are 10.9% and 6.63% in inpatients and outpatients with schizophrenia, respectively. Given that lithium is commonly used in clinical practice, it is of great interest to evaluate the effect of lithium on alleviating Akt1-related deficits. Taking advantage of *Akt1*^*+/−*^ mice to mimic genetic deficiency in patients, behavioral impairments were replicated in female *Akt1*^*+/−*^ mice but were alleviated by subchronic lithium treatment for 13 days. Lithium also effectively alleviated the observed reduction in phosphorylated GSK3α/β expression in the brains of *Akt1*^*+/−*^ mice. Furthermore, inhibition of Akt expression using an Akt1/2 inhibitor significantly reduced neurite length in P19 cells and primary hippocampal cell cultures, which was also ameliorated by lithium. Collectively, our findings implied the therapeutic potential of lithium and the importance of the AKT1-GSK3 signaling pathway.

## Introduction

Schizophrenia is a costly and devastating neurodevelopmental disorder that affects 1% of the world’s population^1^. Generally, schizophrenia is characterized by three categories: positive symptoms, negative symptoms, and cognitive deficits. Schizophrenia also shows substantial sex differences in the incidence rate, age of onset, and symptom severity^2–4^. Schizophrenia appears to be a multifactorial disorder with a strong genetic predisposition. Accumulating evidence from human genetic studies suggests that several susceptibility genes or loci, including *AKT1* (PKBα)^5,6^, are involved in the pathogenesis of schizophrenia. The association between schizophrenia and *AKT1* genetic variants was initially reported in a Caucasian family of European descent^7^ and then confirmed in several other ethnic groups^8–12^. Moreover, studies of schizophrenia postmortem brain tissue^7,13^, Akt1-deficient mice^14–19^, and functional neuroimaging in humans^20^ further suggest that the biological function of AKT1 and its mechanism contribute to schizophrenia susceptibility.

AKT1 is a key signaling intermediate downstream of dopamine receptor D2 (DRD2), and the activation of AKT1 and the phosphatidylinositol 3-kinase (PI3K)-AKT-glycogen synthase kinase-3 (GSK3) cascade has been implicated in many neural functions, including neurite outgrowth^21^. GSK3 is a direct downstream serine/threonine kinase of AKT. Convergent evidence suggests that GSK3 is an important mediator of the DRD2-βArr2-AKT-GSK3 signaling pathway^22–24^ and that GSK3 plays critical roles in brain development and schizophrenia pathogenesis^7,25–27^. To further scrutinize the role of AKT1 in the pathogenesis of schizophrenia, the *Akt1* mutant mouse model provides a good gateway with face and construct validity to investigate the cause and effect between Akt1 and schizophrenia. Intriguingly, neither typical nor atypical antipsychotics alleviated behavioral deficits in *Akt1* homozygous knockout female mice, whereas direct or indirect GSK3 inhibitors significantly normalized the observed behavioral impairments in these mice^16^. A pharmacogenetic study also revealed that schizophrenic patients who carried the AKT1 rs1130233 A-allele associated with reduced AKT1 expression had relatively smaller cognitive change compared with non-risk allele carrier patients treated with lithium^28^.

Lithium, a GSK3 inhibitor, was first used in 1949 as a mood-stabilizing drug in the treatment of bipolar disorder or mania^29,30^. Clinically, lithium has also been used for treating severe psychosis symptoms, and lithium alone or lithium augmentation of antipsychotic medications is proposed as an effective treatment for some patients with schizophrenia^24,31^. Moreover, numerous studies show that lithium exerts effects against the models of schizophrenia *in vivo*^22,32^ and *in vitro*^33,34^. Intriguingly, it was reported that lithium had differing cognitive and prefrontal-medial temporal lobe brain outcomes determined by *AKT1* genetic variation in patients with schizophrenia^28^. Emerging evidence indicates that lithium antagonizes dopaminergic neurotransmission and behaviors mediated by the β-arrestin-2/Akt/GSK3 signaling cascade^22,35,36^. Furthermore, lithium attenuates psychostimulant-induced hyperactivity and behavioral sensitization via modulation of dopamine release^37^. Thus, further investigating the effect and therapeutic potential of lithium in *Akt1*-deficient mouse and cellular models is of great interest.

In this study, in Experiment 1, we first performed data mining to reveal and clarify the average utilization rate of lithium in inpatients and outpatients with schizophrenia from the National Health Insurance Research Database (NHIRD) of Taiwan from 2002 to 2013. Given that lithium antagonizes dopaminergic neurotransmission and behaviors mediated by the Akt-Gsk3 signaling pathway and that *AKT1* has been identified as a possible schizophrenia susceptibility gene, we further clarified the effect of lithium in an Akt1 mouse model of schizophrenia and Akt1-deficient neuronal cells. As a complement to human studies, animal and cellular models provide an indispensable and practical approach to elucidate causal relationships between genetic deficits and functions. Taking advantage of the *Akt1* heterozygous mutant (*Akt1*^*+/−*^) mouse model of schizophrenia and Akt1-deficient cellular models, we focused on investigating the therapeutic potential of lithium in the alleviation of *Akt1*-related deficits. In Experiment 2 A, a set of three behavioral tasks was conducted to assess the therapeutic effect of subchronic lithium treatment in the amelioration of behavioral deficits in *Akt1*^*+/−*^ female mice based on previous studies in Akt1 homozygous knockout mice^16,17^. Then, in Experiment 2B, the levels of Gsk3 protein expression in the brain were further measured in another batch of *Akt1*^*+/−*^ female mice after subchronic lithium treatment. In Experiment 3, both AKT1/2 inhibitor and lithium were applied to evaluate whether lithium recovered the modulation of AKT activity on neurite length in P19 cells and primary hippocampal cell cultures. Collectively, our findings supported the therapeutic potential of lithium for the treatment of schizophrenia and the importance of Akt1-Gsk3 as a therapeutic target.

## Results

### Experiment 1: The characterization of the general utilization rate of lithium in patients with schizophrenia from 2002 to 2013 from the NHIRD of Taiwan

The National Health Insurance system of Taiwan was established in 1995. This system covers more than 99.6% of the Taiwanese population (i.e., approximately 23.5 million), and its claims data are released as the NHIRD. The demographic characteristics of clinical medical treatments in inpatients and outpatients with schizophrenia from the NHIRD are shown in Tables 1 and 2, respectively. The four major categories of medications that had been used in all subtypes of DSM-IV diagnosed schizophrenia in inpatients and outpatients recruited from 2002 to 2013 included lithium, haloperidol, olanzapine, and risperidone. As shown in Table 1, for inpatients, among 3365 person-times from 2002 to 2013, lithium was received as adjunctive therapy 367 person-times (10.9%). On average, lithium treatment was received by approximately 30.58 patients per year. For outpatients (Table 2), among 4627 person-times from 2002 to 2013, lithium treatment was received 307 person-times (6.63%). On average, lithium was received by approximately 25.58 patients per year. In consideration of the overall percentage of person-times in each of the three major subtypes of first diagnosed schizophrenia (Table 3), lithium was widely used not only in inpatients with schizoaffective disorder (25.25%) but also in inpatients with the paranoid type of schizophrenia (26.47%) or unspecified schizophrenia (18.38%). Similarly, lithium was used not only in outpatients with schizoaffective disorder (37.46%) but also in outpatients with the paranoid type of schizophrenia (23.45%) or unspecified schizophrenia (7.49%). These results indicate that lithium had been regularly used in both inpatients and outpatients from 2002 to 2013 in our database and that it is used not only in the treatment of schizoaffective disorder but also in other subtypes of schizophrenia.

**Table 1:** The demographic characteristics of clinical medical treatments for inpatients with schizophrenia from the National Health Insurance Research Database (NHIRD) of Taiwan from 2002 to 2013.

	Total inpatients		Lithium		Haloperidol		Olanzapine		Risperidone	
	N = 3365		N = 367 (10.90%)		N = 1716 (51.00%)		N = 981 (29.15%)		N = 1774 (52.72%)	
	Mean	(SD)	Mean	(SD)	Mean	(SD)	Mean	(SD)	Mean	(SD)
Age	41.12	14.34	38.05	10.41	40.37	12.71	40.28	12.75	40.04	13.1
Gender (M:F)	1: 0.97		1: 1.02		1: 0.92		1: 1.02		1: 0.99	
**Distribution by age (N)**										
<18	46		4		15		9		24	
18–24	353		34		147		85		178	
25–34	814		103		443		269		468	
35–44	901		124		527		280		477	
45–54	695		78		371		199		382	
55–65	331		21		135		99		167	
>65	225		3		78		40		78	
**Distribution by year (N)**										
2002	541		51		255		86		182	
2003	414		43		199		105		195	
2004	370		37		167		65		193	
2005	313		31		168		71		160	
2006	278		39		175		69		157	
2007	208		30		124		83		140	
2008	239		31		119		81		163	
2009	241		21		139		88		152	
2010	216		15		108		75		118	
2011	194		24		84		103		111	
2012	183		27		89		83		112	
2013	168		18		89		72		91	

Participating medical care institutions of the NHIRD are required to electronically submit monthly claim documents related to medical expenses. Such documents include information such as patient demographic data, diagnostic codes, medical institutions visited, dates of prescriptions, drugs prescribed, and claimed medical expenses. Individual and hospital identifiers are unique to the NHIRD and cannot be used to trace individual patients or medical care institutions. Our study sample consisted of 3365 inpatients with schizophrenia. The prescription of antipsychotic drugs included lithium, haloperidol, olanzapine, and risperidone. The remaining patients were classified according to the antipsychotic agent that was prescribed to them as of the index date. We also divided the samples into different ages and schizophrenia subtypes to show the demographics.

**Table 2:** The demographic characteristics of clinical medical treatments for outpatients with schizophrenia from the National Health Insurance Research Database (NHIRD) of Taiwan from 2002 to 2013.

	Total outpatients		Lithium		Haloperidol		Olanzapine		Risperidone	
	N = 4627		N = 307 (6.63%)		N = 1462 (31.6%)		N = 913 (19.73%)		N = 1936 (41.84%)	
	Mean	(SD)	Mean	(SD)	Mean	(SD)	Mean	(SD)	Mean	(SD)
Age	42.17	14.96	38.15	10.73	40.83	12.94	40.23	13.54	40.8	14.21
Gender (M:F)	1: 0.96		1: 1.012		1: 0.87		1: 0.93		1: 0.96	
**Distribution by age (N)**										
<18	56		5		13		9		24	
18–24	439		28		115		90		194	
25–34	1104		83		376		259		526	
35–44	1175		102		436		251		484	
45–54	960		67		314		161		381	
55–65	511		19		139		96		199	
>65	380		3		69		47		128	
**Distribution by year (N)**										
2002	851		44		233		93		194	
2003	556		37		155		117		211	
2004	480		30		131		84		206	
2005	410		24		136		65		171	
2006	346		33		123		56		178	
2007	315		29		105		60		145	
2008	288		15		98		67		169	
2009	322		22		126		92		172	
2010	289		18		109		62		124	
2011	277		19		85		68		122	
2012	260		23		94		74		128	
2013	223		13		67		75		116	

Our study sample consisted of 4627 outpatients with schizophrenia from the NHIRD. The methodological details were described previously in Table 1.

**Table 3:** The overall percentage of person-times for the four major categories of medications used in each of the three major subtypes of first diagnosed schizophrenia in inpatients and outpatients.

Total		Lithium		Haloperidol		Olanzapine		Risperidone	
Subtypes	(%)	Subtypes	(%)	Subtypes	(%)	Subtypes	(%)	Subtypes	(%)
**Inpatients**									
Paranoid type schizophrenia	31.08	Paranoid type schizophrenia	26.47	Paranoid type schizophrenia	35.81	Paranoid type schizophrenia	33.63	Paranoid type schizophrenia	36.89
Unspecified schizophrenia	20.66	Schizoaffective disorder	25.25	Unspecified schizophrenia	20.88	Unspecified schizophrenia	22.25	Unspecified schizophrenia	22.1
Simple type schizophrenia	5.46	Unspecified schizophrenia	18.38	Schizoaffective disorder	7.7	Schizoaffective disorder	8.14	Schizoaffective disorder	6.38
Schizoaffective disorder	5.46								
**Outpatients**									
Paranoid type schizophrenia	28.66	Schizoaffective disorder	37.46	Paranoid type schizophrenia	30.3	Paranoid type schizophrenia	33.08	Paranoid type schizophrenia	36.57
Unspecified schizophrenia	14.39	Paranoid type schizophrenia	23.45	Unspecified schizophrenia	18.88	Unspecified schizophrenia	15.01	Unspecified schizophrenia	14
Simple type schizophrenia	5.9	Unspecified schizophrenia	7.49	Schizoaffective disorder	7.87	Schizoaffective disorder	7.56	Schizoaffective disorder	5.99
Schizoaffective disorder	5.14								

The methodological details were described previously in Table 1.

### Experiment 2 A: Subchronic injections of lithium alleviated behavioral deficits in *Akt1*^*+/−*^ female mice

**Figure 1:**
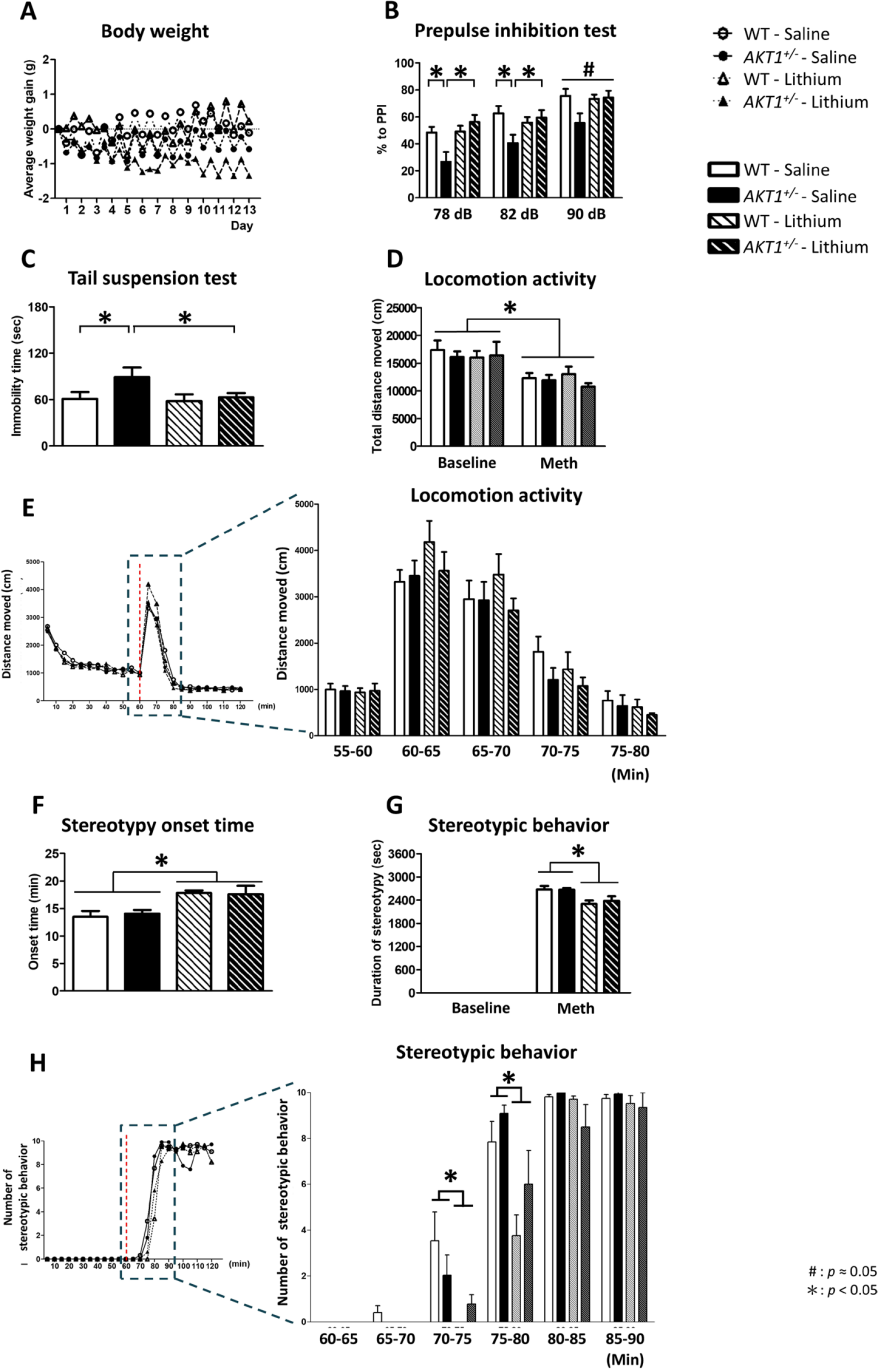
The effect of subchronic lithium treatment on the amelioration of behavioral deficits (mean + SEM) in WT and *Akt1*^*+/−*^ female mice in Experiment 2 A. (**A**) Average weight gain throughout the 13 days of lithium (100 mg/kg, i.p.) or vehicle administration. (**B**) Prepulse inhibition (PPI) was measured on the 9^th^ day after subchronic lithium/vehicle administration. (**C**) Tail suspension test was conducted 11 days after subchronic lithium/vehicle administration. (**D**) Methamphetamine (Meth, 10 mg/kg, i.p.)-induced locomotion and stereotypic behavior were evaluated on the 13^th^ day after subchronic lithium/vehicle administration. (**E**) The effect of methamphetamine on the short term and long term locomotion (i.e., 0–20 and 20–60 minutes after Meth injection, respectively) across 5-minute time bins among the 4 groups. The red dashed line indicates the time of methamphetamine injection. (**F**) The onset time of Meth-induced stereotypic behaviors was recorded. (**G**) The duration of Meth-induced stereotypic behaviors was recorded in an open field. Baseline: 0–60 min; Meth: 60–120 min. (**H**) The effect of methamphetamine on the total number of methamphetamine-induced stereotyped behavior across 5-min time bins among the 4 groups. The red dashed line indicates the time of methamphetamine injection. Methamphetamine was injected after the end of the 60-min baseline. n = 10 per group. *p < 0.05; #*p* ≈ 0.05.

As shown in Fig. 1A, no significant group differences were found in body weight gain across the 13-day subchronic injections of lithium or saline (all *p* > 0.05). A set of three behavioral tasks was conducted to assess the therapeutic effect of subchronic lithium treatment in the amelioration of behavioral deficits observed in female *Akt1*^*+/−*^ mice. As depicted in Fig. 1B, compared to WT littermate controls, female *Akt1*^*+/−*^ mice exhibited a profound behavioral deficit in acoustic prepulse inhibition (PPI). There were significant main effects on the genotype (78 dB: F(1,36) = 8.085, *p* < 0.05) and genotype X treatment interaction (78 dB: F(1,36) = 7.377, *p* < 0.05; 82 dB: F(1,36) = 5.638, *p* < 0.05; 90 dB: F(1,36) = 3.784, *p* = 0.06). Statistical analysis further revealed significant differences in the simple main effects of genotype in the saline treatment (*p* < 0.05) and treatment differences in *Akt1*^*+/−*^ female mice (*p* < 0.05). Fisher’s least significant difference (LSD) post hoc analysis indicated that female *Akt1*^*+/−*^ mice displayed a significant reduction in PPI, which was normalized by subchronic injections of lithium, especially at 78 and 82 dB (*p* < 0.05). Furthermore, as shown in Fig. 1C, there was a significant difference in immobility time in *Akt1*^*+/−*^ female mice in the tail suspension test compared to that in their WT littermate controls (t(18) = 1.976, *p* < 0.05). Subchronic injections of lithium had no effect on WT controls but significantly alleviated the observed behavioral deficits in *Akt1*^*+/−*^ female mice (t(18) = 1.960, *p* < 0.05).

For methamphetamine-induced locomotion, the injection of 10 mg/kg methamphetamine resulted in a significant reduction of locomotion in all groups, but no significant difference among the 4 groups was found before or after methamphetamine injection (Fig. 1D). For the onset time of methamphetamine-induced stereotypic behavior, there was a significant main effect of treatment (F(1,36) = 14.868, *p* < 0.05). We further analyzed the effect of methamphetamine on the short-term and long-term locomotion (i.e., 0–20 and 20–60 minutes after Meth injection, respectively) across 5-minute time bins. As shown in Fig. 1E, lithium-treated *Akt1*^*+/−*^ mice did not exhibit any differential response or effect to methamphetamine-induce hyperlocomotion in both short (i.e., 20 min after inject) and long (20–60 min after inject) terms. Furthermore, as depicted in Fig. 1F, mice that received lithium injections delayed onset time than did mice treated with saline. However, there was no genotypic difference under each treatment. Similarly, as depicted in Fig. 1G, there was also a significant main effect of treatment in the duration of methamphetamine-induced stereotypic behavior (F(1,36) = 13.115, *p* < 0.05). Mice that received lithium injections spent less time in methamphetamine-induced stereotypic behavior than did mice treated with saline. Additionally, no significant genotypic differences were found among the treatments. We further analyzed the effect of methamphetamine on the total number of stereotypic behaviors across 5-minute time bins after methamphetamine injection. As shown in Fig. 1H, significant treatment effects were revealed 75–80 and 80–85 min after methamphetamine injection (F(1,36) = 57.520, *p* < 0.05 and F(1,36) = 13.075, *p* < 0.05, respectively). Thus, our results of onset time, duration, and frequency support the treatment effect of lithium on the alleviation of methamphetamine-induced stereotypic behaviors in mice.

### Experiment 2B: Subchronic injections of lithium enhanced phosphorylated Gsk3α at serine 21 (pGsk3α) and phosphorylated Gsk3β at serine 9 (pGsk3β) expressions in the mouse brain

**Figure 2:**
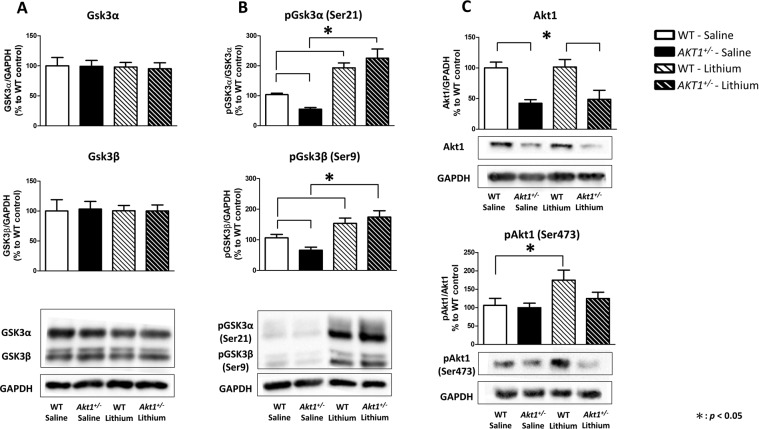
The effect of subchronic injections of lithium or saline on phosphorylated Gsk3α/β (pGsk3α/β at Ser21/Ser9) and phosphorylated Akt1 (pAkt1 at Ser473) expressions in the brains of WT and *Akt1*^*+/−*^ female mice in Experiment 2B. (**A**) Quantitative results (mean + SEM) of Gsk3α and Gsk3β expression in the brains of WT and *Akt1*^*+/−*^ female mice after lithium or vehicle injection. Representative Western blot images of Gsk3α (51 kDa), Gsk3β (46 kDa), and GAPDH (37 kDa). (**B**) Quantitative results (mean + SEM) of pGsk3α (Ser21) and pGsk3β (Ser9) expression in the brains of WT and *Akt1*^*+/−*^ female mice after lithium or vehicle injection. Representative Western blot images of pGsk3α (Ser21, 51 kDa), pGsk3β (Ser9, 46 kDa), and GAPDH (37 kDa). (C) Quantitative results (mean + SEM) of Akt1 and pAkt1 (Ser473) expression in the brains of WT and *Akt1*^*+/−*^ female mice after lithium or vehicle injection. Representative Western blot images of Akt1 (60 kDa), pAkt1 (Ser473, 60 kDa), and GAPDH (37 kDa). n = 5 per group, *p < 0.05.

To confirm the effect of subchronic lithium injections on the mouse brain, we evaluated the levels of Gsk3α/β and the expressions of pGSK3α/β (Ser21/9) in whole mouse brain using Western blotting. As shown in Fig. 2A, no significant group difference was found in the expression of Gsk3α and Gsk3β (*p* > 0.05). In contrast, for the levels of pGsk3α/β expression (Fig. 2B), there were significant main effects of treatment (pGsk3α: F(1,16) = 58.091, *p* < 0.05; pGsk3β: F(1,16) = 18.675, *p* < 0.05) and the genotype x treatment interaction (pGsk3α: F(1,16) = 9.393, *p* < 0.05; pGsk3β: F(1,16) = 7.785, *p* < 0.05). Statistical analysis further revealed significant differences in the simple main effects of genotype in the saline-treated groups (*p* < 0.05) and treatment effect in both WT and *Akt1*^*+/−*^ female mice (all *p* < 0.05). Fisher’s protected least significant difference (LSD) post hoc analysis indicated that compared to their WT littermate controls, *Akt1*^*+/−*^ female mice had a significant reduction in pGsk3α/β (Ser21/Ser9). Thus, lithium treatment significantly increased pGsk3α/β (Ser21/Ser9) levels in both *Akt1*^*+/−*^ and WT female mice and effectively alleviated the observed pGsk3α/β (Ser21/Ser9) reduction in *Akt1*^*+/−*^ female mouse brains (all *p* < 0.05). In addition, we further evaluated the effect of lithium on total Akt1 expression and the activation of Akt1 (pAkt1 at serine 473) in the mouse brains. As shown in Fig. 2C, the injections of lithium had no effect on total Akt1 expression but significantly increased Akt1 phosphorylation on serine 473 (pAkt1) in WT mice compared to saline-treated WT mice (t(8) = 2.380, *p* < 0.05). This result indicated that lithium enhanced pAkt1 in the brain of WT mice but had less effect in *Akt1*^*+/−*^ mice.

### Experiment 3: The effect of lithium on the amelioration of AKT1/2 inhibitor-induced neuronal defects on neurite length was confirmed in P19 cells and primary hippocampal cell cultures

**Figure 3:**
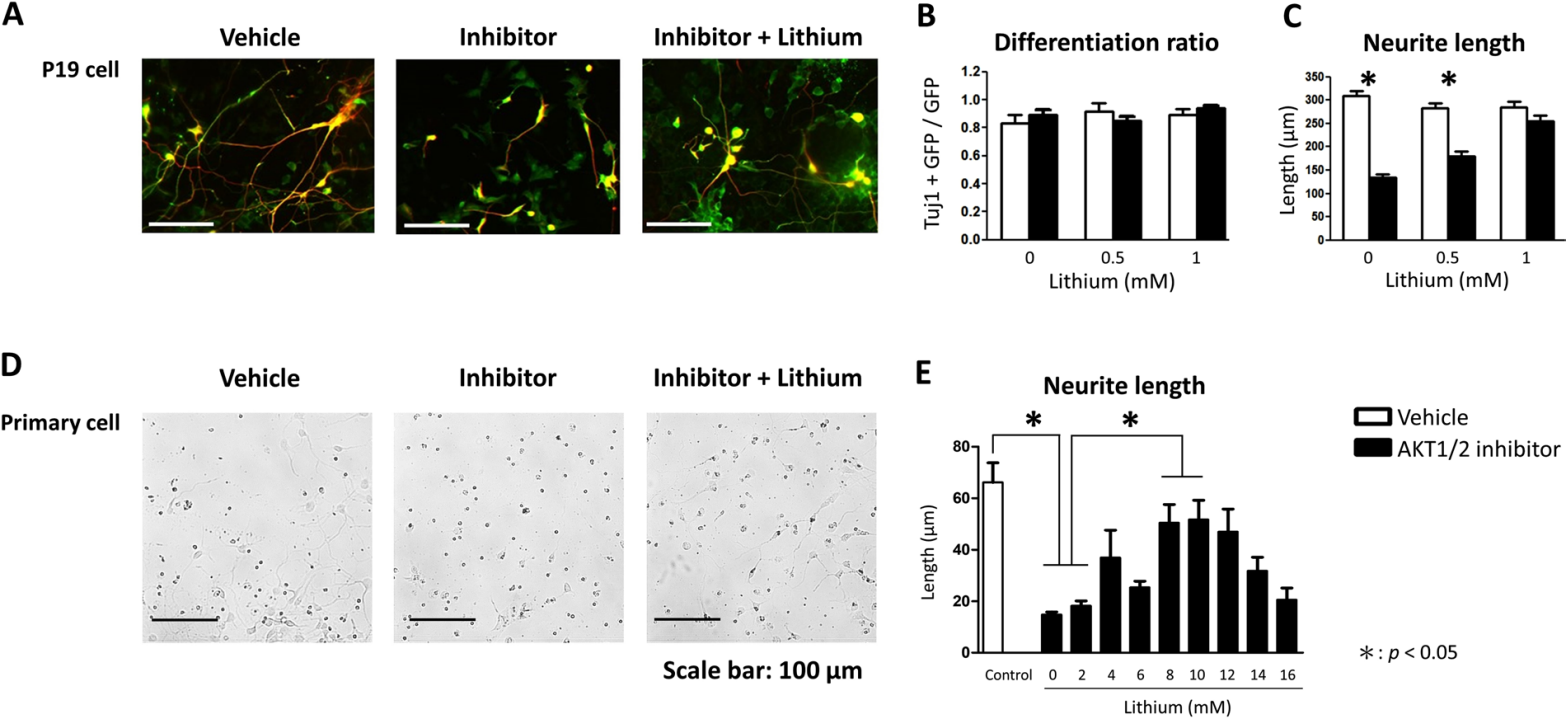
The effect of lithium on the amelioration of AKT1/2 inhibitor-induced neuronal defects on neurite length (mean + SEM) was confirmed in P19 cells and primary hippocampal cell cultures in Experiment 3. (**A**) A panel of representative images of GFP (green; for transfected cells)-stained P19 cells and Tuj1 (red; for differentiated neurons)-positive P19 cells treated with either vehicle, AKT1/2 inhibitor, or AKT1/2 inhibitor + lithium. (**B**) Neither AKT1/2 inhibitor nor lithium had any significant effect on the proportion of differentiated neuron-like cells from P19 cells (Tuj1 + GFP/GFP). Tuj1 is a neuronal marker for Ascl1-induced differentiation of P19 cells into neuron-like cells. GFP is a transfection marker. (**C**) In P19 cells, AKT1/2 inhibitor induced a reduction in neurite length, which was alleviated by 1.0 mM lithium. (**D**) A panel of representative bright-field microscopy images of primary cultures of hippocampal neurons treated with either vehicle, AKT1/2 inhibitor, or AKT1/2 inhibitor + lithium. (**E**) The effective dose of lithium on the amelioration of AKT1/2 inhibitor-induced decrease in neurite length was further evaluated in primary hippocampal cell cultures. *p < 0.05.

To further examine the effect of lithium on neuronal functions, we used both P19 cells and primary hippocampal cell cultures treated with AKT1/2 inhibitor as i*n vitro* Akt-deficient neuronal models in this experiment. A panel of representative images of P19 cells treated with either vehicle, AKT1/2 inhibitor, or AKT1/2 inhibitor + lithium is shown in Fig. 3A. As depicted in Fig. 3B, compared to the vehicle control group, neither the AKT1/2 inhibitor nor lithium group showed any significant effect on Ascl1-induced differentiation of P19 cells into neuron-like cells (all *p* > 0.05). In contrast, there was a significant interaction between lithium treatment and AKT1/2 inhibitor on neurite length (F(2, 1046) = 26.444, *p* < 0.05, Fig. 3C). Statistical analysis further revealed that the AKT1/2 inhibitor induced a significant reduction in neurite length, which was normalized by 1.0 but not 0.5 mM lithium.

The effect of lithium on neuronal functions was further confirmed in primary hippocampal cell cultures in this experiment. A panel of representative images of primary hippocampal cell cultures treated with either vehicle, AKT1/2 inhibitor, or AKT1/2 inhibitor + lithium is shown in Fig. 3D. As shown in Fig. 3E, there was an overall significant group difference in neurite length (F(9,123) = 4.965, *p* < 0.05). Compared to the vehicle control group, the AKT1/2 inhibitor group showed a significant reduction in neurite length (t(18) = 11.299, *p* < 0.05). AKT1/2 inhibitor-induced reduction resulted in an inverted U-shaped dose-response to lithium. Among the dosages tested in this experiment, only 8 and 10 mM lithium significantly ameliorated AKT1/2 inhibition-induced reduction in neurite length in primary hippocampal cell cultures (8 mM: t(27) = 5.092; 10 mM: t(28) = 4.832, both *p* < 0.05).

## Discussion

Some patients with schizophrenia do not achieve treatment satisfaction with current typical/atypical antipsychotics, and various add-on medications have inevitably been used. Positive evidence from clinical studies and meta-analysis demonstrates that lithium is used as a mood stabilizer and is effective for some schizophrenia-related patients^31,38,39^. In daily clinical practice, lithium appears to be used in a certain percentage of patients with schizophrenia for augmentation of neuroleptic efficacy on their psychotic symptoms, treatment of aggression and excitement, and treatment of affective symptoms^31,40^. However, to date, there is no solid evidence from clinical studies to indicate that lithium is an effective standalone treatment for patients with schizophrenia. Conflicting reports indicate that combination treatment with lithium and antipsychotics is somewhat useful in treating schizophrenia in some studies^41–43^ but not in other studies with better trial design^44,45^.

In this study, we first performed data mining from the NHIRD of Taiwan and provided historical insurance data to characterize the general utilization rate of lithium in patients with schizophrenia. Among the patients diagnosed with schizophrenia recruited in the database from 2002 to 2013, 10.9% of inpatients and 6.63% of outpatients received lithium as a part of their treatments. As far as we know, there is no similar database or report like ours, especially on such a large scale. However, there are a few studies reporting the use of lithium in either schizophrenic inpatients or outpatients separately. For example, the prescription data of schizophrenic inpatients from the database of the Drug Safety Program in Psychiatry (AMSP) showed that lithium was used in 2.1% of total inpatients^46^. And the US national Medicaid data revealed that mood stabilizer (including lithium and other antiepileptic) were administered to 15% of adult schizophrenic outpatients who also treated with a single antipsychotic^47^. The ratio might vary from one to another study. But the key conclusions from these studies agree with each other and suggest that lithium is commonly used in clinical practice for patients with schizophrenia. Findings from our data mining are highly consistent with these studies. Notably, in our current study, we also found that lithium was widely used not only in patients with schizoaffective disorder but also in patients with the paranoid type of schizophrenia or unspecified schizophrenia. One possible explanation is that mood-stabilizing drugs, such as lithium, are most often used to treat the symptoms of bipolar disorder and patients with other illnesses such as schizoaffective disorder, may also benefit from them. It was reported that lithium is widely used in clinical practice for people with schizoaffective disorders, although there is no evidence to support this use^31^. Our current study provides a plausible mechanism for the effect of lithium in the treatment of symptoms of schizophrenia and schizoaffective disorder. Thus, our data mining results indicate and confirm that lithium has been regularly used in both inpatients and outpatients and that lithium might be beneficial for the treatment of schizoaffective disorder and other subtypes of schizophrenia.

Arguably, schizophrenia is a multifactorial disorder with a strong genetic predisposition. Intriguingly, it was reported that schizophrenic patients with AKT1 genetic variation influenced the medial temporal lobe development and cognitive function and lithium had differing cognitive and prefrontal-medial temporal lobe brain outcomes determined by *AKT1* genetic variation in these patients^28^. Although it is nearly impossible to recapitulate the full phenotypic spectrum of schizophrenia in any animal, animal models provide indispensable tools for testing drug effects in detail and elucidating the underlying mechanisms^48^. Given the involvement of AKT1 in the pathogenesis of schizophrenia^7,13^ and the importance of AKT in the DRD2-PI3K-AKT-GSK3 signaling pathway^22–24^, both *in vivo* and *in vitro* Akt-deficient models were applied to investigate the therapeutic potential of lithium, a GSK3 inhibitor, on the regulation of functional deficits in Experiments 2 and 3. In Experiment 2, the use of genetically modified mice that carry an alteration in the *Akt1* gene as an experimental tool offers an alternative model that mimics an *AKT1* deficiency in some patients with schizophrenia^7,13^ and provides a feasible model to characterize the forward and reverse translation approaches^49^. As reported in our previous study in *Akt1*^*−/−*^ female mice^16^, our behavioral data replicated that *Akt1*^*+/−*^ female mice also exhibited similar behavioral deficits in acoustic PPI and tail suspension tests. Similar to *Akt1*^*−/−*^ female mice, *Akt1*^*+/−*^ female mice and their littermate controls exhibited no genotypic difference in their spontaneous locomotor activity. Intriguingly, subchronic administration of lithium significantly ameliorated the observed behavioral deficits in acoustic PPI and tail suspension tests in *Akt1*^*+/−*^ female mice. This treatment also alleviated the onset time, total duration, and total number of methamphetamine-induced stereotypy in both *Akt1*^*+/−*^ and WT female mice.

In agreement with our behavioral data in Experiment 2, alterations in pGsk3α/β (Ser21/Ser9) and pAkt1 (Ser473) expression were further confirmed in the mouse brain one hour after lithium injection. Compared to WT littermate controls, *Akt1*^*+/−*^ female mice had a significant reduction in pGsk3α/β (Ser21/Ser9) in the brain. After subchronic lithium injection, pGsk3α/β (Ser21/Ser9) expressions were significantly increased in both *Akt1*^*+/−*^ and WT female mice. As previously reported, lithium directly and indirectly inhibits Gsk3 via increasing the regulatory amino-terminal domain phosphorylation of GSK3^22,50,51^. However, our results also revealed that lithium activated pAkt1 (Ser473) in WT females but not in female *Akt1*^*+/−*^ mice. It evidenced that lithium directly and indirectly inhibited Gsk3 activity in WT mice but only directly inhibited Gsk3 in *Akt1*^*+/−*^ mice. These results implied that the potential therapeutic target for schizophrenia patients with AKT1 deficiency should focus on AKT1 downstream cascades and the AKT1-GSK3 signaling pathway is an appropriate objective for future translation researches. In line with the observed behavioral amelioration in *Akt1*^*+/−*^ female mice treated with lithium, the injection of lithium increased pGsk3α/β (Ser21/Ser9) expression in WT controls as well as effectively alleviated the observed reduction in pGsk3α/β (Ser21/Ser9) in the *Akt1*^*+/−*^ female mouse brain. Although we did not specifically identify the distinct functions of Gsk3α and Gsk3β in this study, convergent evidence from previous studies implicates the involvement of *GSK3β* in the symptoms of neuropsychiatric disorders^52–54^. For example, continuous expression of Gsk3β in the hippocampal dentate gyrus of mice reportedly induced pro-depressant-like effects in the tail suspension test^55^. Gsk3β activity in the frontal cortex, a region central to the neurocircuitry modulating PPI^56^, is positively correlated with PPI responses among different strains of mice^57^. In addition, accumulating data show that *Gsk3*^*+/−*^ mice exhibit a markedly blunted response to acute amphetamine and decreased immobility time in the forced swim test^22,58^, another common behavioral assay for depressive-like behavior in rodents. Furthermore, abundant evidence suggests that both acute and chronic lithium administration mediates Akt-Gsk3 signaling in response to behavioral activity, such as depressive-like behavior, sensorimotor gating function, dopamine-dependent responses and other schizophrenia-related behaviors^22,59–62^. Along with previous findings, our current results in *Akt1*^*+/−*^ female mice suggest the importance of AKT1-GSK3 signaling in schizophrenia and treatment selection.

In addition, emerging *in vitro* evidence indicates the involvement of AKT-GSK3 signaling in neurogenesis, neural polarization, and neurite outgrowth^63,64^. Taking advantage of Akt-deficient neuronal models, we further examined the effect of lithium on neuronal functions in Experiment 3. The application of AKT1/2 inhibitor did not affect neuronal differentiation of P19 cells into neuron-like cells, consistent with previous findings regarding Akt1 deficiency for overall neuronal differentiation^14,65^. As previously reported in different cell types and *Akt1*^*−/−*^ mice, AKT (or Akt1) deficiency resulted in a reduction in neurite outgrowth^16,17,19,64^. Our *in vitro* data in P19 cells and primary hippocampal cell cultures are in line with these previous findings and further indicated that lithium is capable of alleviating the observed impairments in neurite length. Although lithium exerts its neuroprotective effect via inhibition of GSK3^66,67^, inhibition of GSK3 activity through lithium also impairs neurite growth in native cultures in a dose-dependent manner^68,69^. As demonstrated in Experiment 3, optimal doses of lithium are needed for the rescue of AKT1/2 inhibitor-induced defects in neurite length in primary hippocampal cells. The narrow therapeutic index of lithium increases the risk of toxicity, and lithium overdose induces acute illnesses^70^. Nevertheless, the therapeutic potential of lithium on synaptic connectivity and neuromorphology is worthy of further investigation, especially during neuronal development.

In summary, in agreement with the common impression of lithium use in the treatment of some patients with schizophrenia, our data mining results from the NHIRD clearly demonstrated that the average utilization rates of lithium were 10.66% in inpatients with schizophrenia and 6.63% in outpatients with schizophrenia. Despite some obvious limitations in the use of the NHIRD, our data indicated that lithium was widely used in patients with schizoaffective disorder as well as in patients with the paranoid type of schizophrenia or unspecified schizophrenia. Taking advantage of the Akt1 mutant mouse model of schizophrenia and Akt-deficient cellular models, we confirmed that Akt deficiency resulted in behavioral deficits in *Akt1*^*+/−*^ female mice and neuromorphological changes in cell models. Importantly, we further demonstrated the therapeutic effect of lithium in the amelioration of behavioral deficits in *Akt1*^*+/−*^ female mice and reduced neurite length in P19 cells and primary hippocampal cell culture. Our findings support the importance of the AKT-GSK3 signaling pathway and the therapeutic potential of lithium in the treatment of schizophrenia-related phenotypes. Nonetheless, further studies are greatly needed.

## Materials and Methods

### Experiment 1: Characterizing utilization rate of lithium in patients with schizophrenia from the NHIRD of Taiwan

#### Data source and study subjects

Experiment 1 is based on data from the NHIRD of Taiwan. The protocol for Experiment 1 was approved by the Institutional Review Board (IRB) of National Taiwan University Hospital, Taipei, Taiwan. Patient records/information were anonymized and deidentified prior to analysis, and the need for written informed consent was waived by the IRB. We included all patients in the NHIRD who had been diagnosed with schizophrenia-spectrum disorder and were prescribed at least one dose of an antipsychotic drug between January 1, 2002, and December 31, 2013. A patient with schizophrenia-spectrum disorder was defined as an inpatient/outpatient with at least one inpatient or outpatient record pursuant to the International Classification of Diseases, Ninth Revision, Clinical Modification (ICD-9-CM) code 295.x. Our study sample consisted of 3365 inpatients and 4627 outpatients with schizophrenia from the NHIRD. The prescription of antipsychotic drugs whose brand-name and generic forms are both available in Taiwan was traced from NHI claim records, including lithium, haloperidol, olanzapine, and risperidone. We also divided the samples into different ages and schizophrenia subtypes to show the demographics for inpatients and outpatients in Tables 1 and 2, respectively.

### Experiment 2: Assessing the therapeutic effect of lithium in the amelioration of behavioral deficits in the *Akt1*^*+/−*^ mouse model of schizophrenia (Experiment 2 A) and examining the levels of Gsk3α/β and pGsk3α/β (Ser21/Ser9) expression in the mouse brain after lithium injection (Experiment 2B)

#### Animals

All female *Akt1*^*+/−*^ and WT littermate mice used in this study were generated from *Akt1*-heterozygous breeding pairs backcrossed onto a C57BL/6 J background over >10 generations as described previously^14–19^. All animals were 90–100 days old at the beginning of the experiment. Animals were housed individually at least one week before experimental testing. The minimum number of mice was used in accordance with the 3 R principle of animal use. The entire animal procedures were performed according to protocols approved by the Animal Care and Use Committee established by the National Taiwan University.

#### Subchronic lithium and saline administration

Two batches of drug-naïve *Akt1*^*+/−*^ female mice and their WT littermate controls were used to evaluate behavioral performance and protein expression after subchronic lithium or saline administration. All mice were handled and weighed daily at least 1 week before the administration. According to previous studies^71,72^, each mouse received either lithium (Sigma, St Louis, MO, USA; 100 mg/kg, *i.p*., dissolved in saline) or vehicle twice per day at 9:00 am and 9:00 pm and underwent 13 days of lithium administration (n = 10 per group). On the 9^th^, 11^th^, and 13^th^ days, behavioral testing was conducted in the first batch of mice one hour after lithium/saline administration. All mice in the second batch (n = 5 each) also received subchronic injections of lithium or saline, and they were sacrificed one hour after the second injection on the 9^th^ day.

#### Behavioral testing

In Experiment 2 A, a set of three behavioral tasks (including PPI, tail suspension test, and methamphetamine-induced stereotypic behavior) were performed in sequence. These experimental tasks were conducted to evaluate sensorimotor gating function, depressive-like behavior, and psychomotor agitation, respectively. Behavioral testing was performed during the dark cycle. The details of each behavioral test are briefly described as follows.

The PPI of the startle reflex represents the sensorimotor gating function and shows the core feature of schizophrenia in humans and animal models^73,74^. To assess the sensorimotor gating function, we tested each mouse with an SR-LAB startle apparatus (San Diego Instruments, San Diego, CA, USA). As previously described^16^, the same experimental procedures were applied and conducted. The tail suspension test was used to assess depressive-like behaviors as a model of negative symptoms of schizophrenia^75,76^. The behavioral performance of each mouse was subsequently scored during the 6 min tail suspension period with a digital video camera. Immobility was defined as when the mouse’s four paws, body, head, and tail were all immobile. The details of the experimental setup have also been described previously^16^.

Methamphetamine-induced locomotion and stereotypic behavior were modified and conducted as previously described^77^. Briefly, to assess methamphetamine-induced stereotypic behavior and spontaneous locomotor activity, we placed each mouse into an open-field apparatus (25.40 × 25.40 × 40.64 cm^3^, Coulbourn Instruments, Whitehall, PA, USA) under dim lighting conditions (60 lx). Motor activity parameters (total travel distance moved) were monitored and recorded over a 1-hour baseline period and a 1-hour drug period using a SMART video tracking system (Panlab, Harvard Apparatus, USA). After a 1-hour baseline period of spontaneous locomotor activity, all animals were also observed and recorded for stereotypy (including both the onset time and the duration of stereotypy) following a high dose of methamphetamine administration (10 mg/kg, *i.p*., dissolved in saline; from Food and Drug Administration, Department of Health, Taipei, Taiwan). Since individual behaviors were unchanged for long periods (>30 s) after methamphetamine treatment, it was recorded by hand and defined as stereotypic behaviors. We scored and calculated the onset time, total duration, and the number of stereotypic behavior after methamphetamine injection. Any continued stereotyped behavior that was maintained for over 30 sec was considered as a new behavioral event during our behavioral testing. The stereotypic behaviors include inactivity (awake, inactive, and sleeping), ambulation, rearing, persistent locomotion, head bobbing (up-and-down movements of the head), continuous sniffing, circling, and continuous nail and/or wood chip biting or licking, according to a previously described method^78^.

In Experiment 2B, based on the behavioral performance, Western blotting was utilized in a new batch of drug-naïve mice to examine the expression of phosphorylated Gsk3α (pGsk3α at serine 21), phosphorylated Gsk3β (pGsk3β at serine 9), and phosphorylated Akt1 (pAkt1 at serine 473) in the whole brain (excluding cerebellum) of adult female *Akt1*^*+/−*^ mice and their WT littermates (n = 5 each). The mouse brain was harvested one hour after lithium injection on day 9 and stored at −80°C before use. After sample preparation, Western blotting was performed and the blot was probed with the following antibodies: Gsk3α/β (1:1000, #5676), pGsk3α/β (Ser21/Ser9, 1:1000, #8566), Akt1 (1:1000, #2967), pAkt1 (Ser473, 1:200, #9018) and GAPDH (1:5000, #2118) (Cell Signaling Technology, Inc., Danvers, MA, USA). Immune complexes were shown using the appropriate peroxidase-conjugated secondary antibodies (Cell Signaling Technology). Bound antibody was detected using an enhanced chemiluminescence (ECL) kit (Millipore), and densitometric analysis was performed using ImageJ (Java-based image processing program developed at the National Institutes of Health). The experimental details were described previously^14^.

### Experiment 3: Evaluating the effect of lithium on the amelioration of AKT1/2 inhibitor-induced defects on neurite length in P19 cells and primary hippocampal cell cultures

#### P19 cells

To further investigate the effect of lithium on the regulation of AKT1/2 inhibitor-induced defects on neuron differentiation and neuron outgrowth, we used P19 mouse embryonal carcinoma cells (P19 cells) treated with AKT1/2 inhibitor as an Akt-deficient neuronal model as previously described^14^. Briefly, mouse P19 cells from National Taiwan Normal University, Taipei, Taiwan, were maintained in αMEM medium supplemented with 100 units/ml penicillin, 100 μg/ml streptomycin, 7.5% fetal bovine serum (FBS), and 2.5% calf serum^79^. The initial seeding density of the cells is 1.2 × 10^5^ cells in 1 ml per well (in a 12-well plate). The cell density should reach 90–95% prior to transfection. For neuronal differentiation, P19 cells were transfected with the US2-Ascl1 vector to induce neural differentiation; additionally, AKT1/2 inhibitor (1 μM, Calbiochem, San Diego, CA, dissolved in DMSO) or lithium (0, 0.5 and 1 mM, Sigma, St. Louis, MO, USA, dissolved in distilled water) was mixed into the medium during DIV 0–3. To measure the differentiation ratio and neurite length of P19 cells, we performed immunocytochemistry at DIV 3 using antibodies against green fluorescent protein (GFP, 1:2000; Molecular Probes) for transfected cells and neuron-specific class III beta-tubulin (Tuj1, 1:1000; Covance) for differentiated neurons. The proportions of P19 cells expressing Tuj1 among all GFP-positive cells were calculated. Microscopic images of stained neuron-like cells were obtained using 20x and 40x objectives by Nikon (Eclipse 80i; Nikon, Tokyo, Japan) with Image-Pro Plus v7.0 (Media Cybernetics, Rockville, MD, U.S.A.). Neuronal density and the length of neurite outgrowth were quantified and calculated using ImageJ (NIH, Bethesda, MD, U.S.A.). Neurite outgrowth was quantified by documenting random fields, measuring the length of the neurites and counting the number of cell bodies. P19 cells have few branches. We measured all neurites from the cell body surface to the end of the neurite in μm and averaged them.

#### Primary hippocampal cell culture

To further examine the effective dose of lithium on the rescue of AKT1/2 inhibitor-induced defects on neurite length, we applied different doses (0, 2, 4, 6, 8, 10, 12, 14, 16 mM) of lithium to primary hippocampal cell cultures. Primary cell cultures were prepared from Sprague-Dawley rat pups on postnatal day 0 or 1. Hippocampi were dissected in petri dishes filled with ice-cold dissection medium comprising Earle’s Buffered Salt Solution (EBSS; with no Ca^+2^ or Mg^+2^ and no phenol red; Invitrogen) and HEPES (10 mM; Sigma). The dissected hippocampi were cut into pieces and dissociated by papain treatment (10 U/ml; Worthington) at 37 °C followed by trituration with flame-polished Pasteur pipettes. Cells were plated on 22-mm coverslips precoated with poly-L-lysine (50 µg/ml; Sigma) and laminin (20 µg/ml; Sigma). Cultures were maintained in serum-free neurobasal medium (Invitrogen) supplemented with B27 (Invitrogen), L-glutamine (0.5 mM, Invitrogen), penicillin/streptomycin (1%, Invitrogen) and kept at 37 °C in 5% CO2. The initial seeding density of the cells is 1.2 × 10^5^ cells in 1 ml per well (in a 9-well device). The drugs (AKT 1/2 inhibitor (1 μM) or lithium) were mixed in the medium during DIV 0–3. Some cells differentiated into neuron-like cells with a simple neurite extension. Immunocytochemistry was processed at DIV 3 for neurite length measurement using an antibody against Tuj1 (1:1000; Covance). Image acquisitions and neurite outgrowth measurements were conducted and obtained in a manner similar to that previously described for P19 cells.

### Statistical analysis

All data are presented as the mean ± SEM in this study. The data were analyzed by two-way repeated measures analysis of variance (ANOVA) and two-sample Student’s t-tests, where appropriate. F-values reaching significant differences were further evaluated by post hoc analysis using Fisher’s LSD test. A significant interaction effect was further analyzed as the simple main effects of genotype differences within each treatment and treatment differences within each genotype. A priori t-tests with Bonferroni adjustments were conducted to answer specific hypotheses. Statistical analyses were performed using SPSS 20 (IBM Corporation). A P-value < 0.05 was considered statistically significant.
